# Autochthonous Dengue Fever Imported to England from Japan, 2014

**DOI:** 10.3201/eid2101.141581

**Published:** 2015-01

**Authors:** Gotaro Kojima

**Affiliations:** Author affiliation: Japan Green Medical Centre, London, UK

**Keywords:** dengue fever, outbreak, Japan, England, viruses, vector-borne infections, mosquitoes

**To the Editor:** Dengue fever, a mosquito-borne disease caused by dengue virus, can be asymptomatic or result in a variety of clinical manifestations, including fever, headache, myalgia, arthralgia, and rash (*1*). Severe cases can cause shock or severe hemorrhage (*1*). During the past 50 years, dengue has become a public health concern worldwide, rapidly spreading geographically, mainly in tropical and subtropical countries (*1*).

Before 2014, the most recent dengue outbreaks in Japan (located in the temperate zone), started in August 1942 on Kyusyu (the southernmost of Japan’s 4 main islands) and recurred every summer until 1945 (*2*). Although no autochthonous dengue fever has been identified since then, a warning case occurred in August 2013, when dengue virus infection was diagnosed in a German traveler who returned from a 2-week trip to Japan (*3*). Reported here is an autochthonous dengue fever case imported from Japan to England in September 2014.

A 33-year-old international male student studying in England traveled to Japan and stayed with a friend in Tokyo during July–September 2014. In late August, an acute high fever (39.7°C), severe headache, retro-orbital pain, malaise, and loss of appetite developed. He did not have cough, sputum, or rhinorrhea. He returned to England 3 days after symptoms developed. On day 5 after symptom onset, the man noticed a scattered papular erythematous rash on the anterior chest wall. His symptoms had improved substantially. One week later, he experienced exacerbation of the same symptoms (again without cough, sputum, or rhinorrhea), which continued for a few days until he sought care 12 days after initial onset. He had no significant medical history or known allergies and had not traveled to any countries other than Japan.

On examination, the man appeared ill and had a high fever (38.5°C). His lungs were clear, but his pharynx was markedly swollen and erythematous with cervical lymphadenopathy. Despite his severe symptoms, results of laboratory tests showed no or only mild elevations: normal leukocyte count and differentials (6,700 cells/mm^3^ [reference 4,000-11,000] with neutrophils 60% and lymphocytes 26%) and slightly elevated C-reactive protein (0.9 mg/dL [reference 0.0–1.0 mg/dL]) and erythrocyte sedimentation rate (14 mm/h [reference 1–10 mm/h]). Liver enzyme levels were elevated: lactate dehydrogenase 623 IU/L (reference 125–243 IU/L), alanine aminotransferase 78 IU/L (reference 0–55 IU/L), aspartate aminotransferase 58 IU/L (reference 5–45 IU/L), and γ-glutamyl transpeptidase 81 IU/L (reference 12–64 IU/L), with normal total bilirubin (1.0 mg/dL [reference 0.2–1.2 mg/dL]). Mild hyponatremia (sodium 132 mmol/L [reference 135–145 mmol/L]) also was noted; otherwise the test results were unremarkable, including normal platelet count and coagulation panel. Serologic tests were positive for anti–dengue virus IgM but negative for anti–dengue virus IgG: viral RNA was not detected.

The patient was hydrated with intravenous fluid and discharged with acetaminophen as needed for fever and pain. He was instructed to avoid nonsteroidal antiinflammatory drugs because of possible bleeding risk. The sore throat resolved within 1 day, and his pain and fever were well controlled with acetaminophen.

This dengue outbreak in Japan started in Yoyogi Park, a public park in the Tokyo metropolitan area. From August 26, 2014, when the first case was identified, through October 30, a total 160 autochthonous dengue fever infections occurred (*4*). The patient reported here stated that the house where he stayed in Japan is a 2-minute walk from Yoyogi Park and that he was bitten multiple times by mosquitos in late August, although he did not enter the park or other implicated parks.

This outbreak has several possible causes. First, *Aedes albopictus* mosquitoes—1 of 2 main vector mosquitoes of dengue virus—are widespread in Japan. Although the other species, *A. aegypti*, has not been established in Japan, it was once identified at Tokyo International Airport (*5*). Second, the worldwide dengue fever incidence has increased exponentially during the past 50 years (*1*), and the number of cases imported to Japan has increased steadily since 1999 (*6*,*7*). Third, increased international travel, trade, and shipping, in addition to global warming, might have contributed to geographic expansion of the vectors.

Why this dengue fever outbreak started from Yoyogi Park remains unknown. One possibility is the popularity of Yoyogi Park, which holds ≈100 events annually, including many international events. In July and August 2014, just before the first case was identified, Yoyogi Park hosted multiple festivals of countries in dengue-endemic regions, including Southeast Asia and South and Central America (http://www.yoyogipark.info/ad2014/). Dengue virus could have been spread from infected visitors by mosquitoes in the park. Any events that include persons from dengue-endemic regions and held where the vectors are prevalent could be the source of spread.

This case highlights the possible risk for a dengue outbreak in countries to which dengue is not endemic but where the vectors are present. and thus the potential exists for travelers to become infected. Physicians need to be aware of the possibility of dengue fever in patients returning from non–tropical/subtropical countries, obtain a full travel history, and keep apprised of the latest epidemic information.
